# Single‐cell transcriptomics reveals cellular heterogeneity and phenotypic transitions of smooth muscle cells in aortic dissection

**DOI:** 10.1002/imt2.70124

**Published:** 2026-04-21

**Authors:** Liang Shao, Fan Hu, Ling‐Na Zhao, Jian‐Ping Luo, Peng‐Tao Zou, Xiu Liu, Shao‐Yi Zheng, Cong Chen, Lin‐Xiong Ye, Yu‐Xuan Zhou, Jiaqi Zhang, Kaidi Jin, Ping Zhang

**Affiliations:** ^1^ Department of Cardiology, the Second Affiliated Hospital University of South China Hengyang China; ^2^ Hengyang Medical School University of South China Hengyang China; ^3^ Department of Cardiology, Jiangxi Provincial People's Hospital The First Affiliated Hospital of Nanchang Medical College Nanchang China; ^4^ Department of Neurology, Jiangxi Provincial People's Hospital The First Affiliated Hospital of Nanchang Medical College Nanchang China; ^5^ Department of Cardiovascular Surgery, Nanfang Hospital Southern Medical University Guangzhou China; ^6^ Department of Cardiology Ganzhou People's Hospital Ganzhou China; ^7^ Department of General Surgery, Pancreatic Disease Center, Ruijin Hospital Shanghai Jiao Tong University School of Medicine Shanghai China; ^8^ Department of Biological Medicines & Shanghai Engineering Research Center of Immunotherapeutics, School of Pharmacy Fudan University Shanghai China; ^9^ Department of Forensic Medicine, School of Basic Medical Sciences Fudan University Shanghai China

## Abstract

We utilized single‐cell RNA sequencing (scRNA‐seq) to investigate cellular heterogeneity and signaling networks in aortic dissection (AD) tissues compared to adjacent normal tissues. The analysis identified five smooth muscle cell (SMC) subtypes, with SMC2 linked to fibrosis and SMC3 associated with inflammation. Thrombus‐positive AD samples showed upregulated angiopoietin‐like 4 (*ANGPTL4*) and increased M2 macrophages, indicating an immunosuppressive microenvironment. Cell‐cell communication analysis revealed a shift in vascular endothelial growth factor A (*VEGFA*) signaling from SMCs to fibroblasts, disrupting vascular homeostasis. In vitro experiments confirmed SMC2‐induced endothelial‐to‐mesenchymal transition and SMC3‐driven inflammatory responses via mitogen‐activated protein kinase (MAPK) pathways. Immunofluorescence validated elevated insulin‐like growth factor binding protein 2 (*IGFBP2*), procollagen‐lysine 2‐oxoglutarate 5‐dioxygenase 2 (*PLOD2*), and *VEGFA* in AD tissues, supporting their roles in matrix remodeling and angiogenesis. These findings highlight SMC phenotypic switching and altered *VEGFA* signaling as key drivers of AD, proposing novel therapeutic targets to restore vascular integrity.

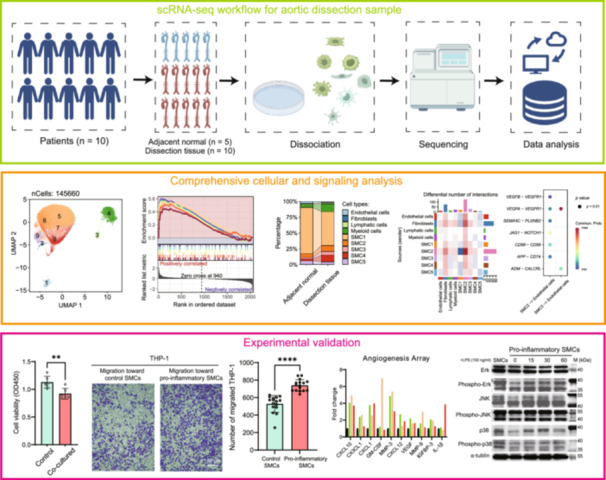

To the Editor,

Aortic dissection (AD) occurs when a tear in the intimal layer of the aorta allows blood to penetrate between the layers of the aortic wall, forming a false lumen. The pathophysiology of AD is primarily driven by the weakening of the medial layer, which is often exacerbated by factors such as hypertension. This weakening facilitates an intimal tear, enabling blood to dissect the aortic layers. Immediate medical or surgical intervention is crucial to prevent life‐threatening complications, including aortic rupture, cardiac tamponade, or organ malperfusion [1, 2]. Despite advancements in diagnostic techniques, surgical approaches, and medical management, AD remains associated with high mortality rates, underscoring the need for a more comprehensive understanding of its underlying mechanisms [3, 4]. Additionally, dissection can affect blood vessels throughout the body, such as the aorta, coronary arteries, and cerebral arteries, further complicating the disease's clinical management.

Previous research has identified genetic predisposition, hypertension, and connective tissue disorders as significant contributors to AD pathogenesis [5, 6, 7, 8]. However, the cellular and molecular mechanisms that drive its initiation and progression remain poorly understood. In this context, the emergence of single‐cell RNA sequencing (scRNA‐seq) has proven invaluable in dissecting cellular heterogeneity and uncovering gene expression changes at unprecedented resolution [9, 10, 11]. Recent scRNA‐seq analyses have revealed notable heterogeneity within cell populations involved in AD, particularly immune cells such as macrophages, smooth muscle cells (SMCs), and endothelial cells (ECs). These studies have demonstrated distinct gene expression alterations in these cell types during AD progression, implicating processes such as inflammation, extracellular matrix (ECM) degradation, and cell death pathways.

While previous scRNA‐seq studies have documented phenotypic heterogeneity among SMCs in AD, most have primarily described subtype composition without dissecting the functional mechanisms underpinning specific phenotypes or their pathological consequences. In particular, the pro‐fibrotic drivers within distinct SMC subsets, the signaling dependencies of inflammatory phenotypes, and the pathological associations between thrombus formation and cellular phenotype, remain poorly understood. To address these gaps, we employed scRNA‐seq to profile AD and adjacent normal tissues, integrating high‐resolution transcriptomic analysis with targeted functional assays to identify key SMC subtypes, delineate their mechanistic roles in fibrosis and inflammation, and explore their interactions with immune and stromal cells. Through this approach, we aim to uncover novel therapeutic targets and provide deeper mechanistic insights into the cellular pathways driving AD progression.

To comprehensively characterize the cellular landscape and molecular alterations in AD, we performed scRNA‐seq on AD lesion tissues from patients with and without intraluminal thrombus, together with paired adjacent normal aortic tissue. Clinical characteristics of the overall study cohort are summarized in Table S1, whereas a complete library‐level sample manifest for all scRNA‐seq libraries is provided in Table S2. scRNA‐seq of AD and adjacent normal tissue profiled 145,660 cells, revealing four major cell types and five SMC subtypes via uniform manifold approximation and projection (UMAP) visualization, with SMCs as the predominant population (Figure 1A). Cell types were identified using canonical markers: myosin heavy chain 11 (*MYH11*) and actin alpha 2 (*ACTA2*) for SMCs, cadherin 5 (*CDH5*) for ECs, and markers for fibroblasts, lymphatic cells, and myeloid cells (Figures 1B, S1A). Comparative analysis revealed marked shifts in SMC subtype distribution in thrombus‐positive samples, with increased SMC3 and SMC4 proportions and near‐complete depletion of SMC2 (Figure 1C).

**FIGURE 1:**
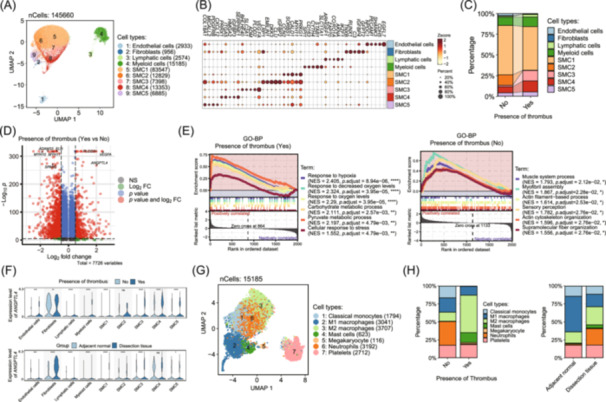
Single‐cell RNA sequencing reveals cellular heterogeneity in thrombus‐related aortic dissection. (A) Uniform Manifold Approximation and Projection (UMAP) plot of all profiled cells, colored by cell type. (B) Heatmap with dot overlay showing the top 5 marker genes for each cell type (Wilcoxon test). (C) Stacked bar plot of the pooled cell‐type composition across 10 aortic dissection (AD) lesion scRNA‐seq libraries, stratified by thrombus status (Yes, *n* = 3; No, *n* = 7). (D) Differential expression analysis of smooth muscle cells (SMCs) between thrombus‐positive and thrombus‐negative AD samples (model‐based analysis of single‐cell transcriptomics test (MAST); Bonferroni‐adjusted *p* values). (E) Gene Ontology Biological Process (GO‐BP) gene set enrichment analysis (GSEA) of SMCs comparing thrombus‐positive versus thrombus‐negative AD samples (Differential expression: MAST; GSEA: false discovery rate [FDR]‐adjusted *q* values). (F) Expression of angiopoietin‐like 4 (*ANGPTL4*) across cell types, stratified by thrombus status (AD samples only, top) and tissue group (bottom) (Wilcoxon test; Benjamini–Hochberg adjustment). (G) UMAP of myeloid cell subtypes. (H) Pooled proportions of myeloid subtypes by thrombus status (AD lesion libraries only, left) and tissue group (10 AD lesion libraries versus 5 paired adjacent normal libraries, right). For all statistical analyses, significance thresholds were set as follows: *p < 0.05; **p < 0.01; ***p < 0.001; ****p < 0.0001.

Within AD specimens, SMCs exhibited distinct gene expression patterns associated with thrombus status (Figure 1D). Thrombus‐positive samples exhibited upregulation of genes such as angiopoietin‐like 4 (*ANGPTL4*), vascular endothelial growth factor A (*VEGFA*), and procollagen‐lysine 2‐oxoglutarate 5‐dioxygenase 2 (*PLOD2*), which are known to play roles in angiogenesis and ECM remodeling. Conversely, genes including elastin (*ELN*), myosin heavy chain 10 (*MYH10*), ADAM metallopeptidase domain 33 (*ADAM33*), and secreted protein acidic and rich in cysteine (*SPARC*) were downregulated, suggesting potential alterations in ECM organization and cellular mechanical programs. We further evaluated the clinical relevance of these differentially expressed genes by relating key representative genes to disease severity features (Figure S1B).

Gene set enrichment analysis (GSEA) of SMCs indicated that thrombus‐positive AD specimens were enriched for hypoxia‐response, metabolic, and cellular stress‐related biological processes, whereas thrombus‐negative specimens were enriched for muscle system processes and cytoskeletal organization programs, including myofibril assembly and actin filament‐based/cytoskeleton organization (Figure 1E). Furthermore, *ANGPTL4* expression showed a broad increase in thrombus‐positive AD specimens across multiple cell populations, with significant differences observed in several SMC subtypes. Notably, SMC2 was nearly absent in thrombus‐positive samples, precluding assessment of *ANGPTL4* expression in this subtype. *ANGPTL4* was also increased in AD (Figure 1F), suggesting a possible link between SMC phenotype and thrombus formation, potentially mediated by macrophage polarization [12, 13] (Figure 1G). M2 macrophages, identified by markers macrophage receptor with collagenous structure (*MARCO*) and *VCAN*, were more abundant in thrombus‐positive and AD tissues compared to normal tissues (Figure 1H). GO Biological Process enrichment across myeloid subtypes showed that M2 macrophages are predominantly enriched in immune‐related pathways (Figure S1C). Together, these results support a cellular framework linking intraluminal thrombus to coordinated alterations in SMC programs and myeloid‐cell polarization, consistent with a thrombus‐associated disease state characterized by hypoxic/stress responses and structural remodeling that may contribute to disease progression.

To investigate cellular composition changes in AD, we compared cell type distributions between AD tissues and adjacent normal tissues. AD tissues demonstrated marked alterations in cellular composition, with notable shifts in SMC populations and immune cell infiltration (Figure 2A). Detailed analysis of SMC populations revealed five transcriptionally distinct subtypes (SMC1–5) with differential abundance between AD and normal tissues (Figure 2B). This phenotypic shift indicates a transition from contractile to synthetic and pathological SMC states during AD progression. Gene‐set enrichment analysis of MSigDB Hallmark and C2 pathways revealed distinct functional programs across the five SMC subtypes (Figure 2C,D). SMC2 exhibited a robust ECM‐remodeling and mesenchymal activation program, with strong enrichment of hallmark and apical junction alongside Reactome extracellular‐matrix organization. By contrast, SMC3 was dominated by hypoxia‐ and inflammation‐driven transcription, marked by Hallmark Hypoxia, tumor necrosis factor A (TNFA) signaling via nuclear factor kappa B (NF‐κB), and inflammatory response, and reinforced by curated hypoxia metagenes (Mense, Winter, Elvidge). In addition, SMC1 preferentially enriched transcriptional and metastasis‐related gene sets, whereas SMC4 showed the strongest activation of MYC proto‐oncogene (*MYC*) targets (Hallmark MYC targets v1/v2 and MYC/MYCN‐related C2 sets). SMC5 displayed a mixed phenotypic profile. At the gene level, SMC2 in AD upregulated key fibrotic mediators, including collagen genes (collagen type I alpha 1 chain (*COL1A1*), collagen type I alpha 2 chain (*COL1A2*), collagen type III alpha 1 chain (*COL3A1*), collagen type IV alpha 1 chain (*COL4A1*)), the matricellular factor cellular communication network factor 2 (*CCN2*), and prostacyclin synthase (*PTGIS*) (Figure S2A). SMC3 downregulated contractile markers (myosin light chain 9 (*MYL9*), *ACTA2*, superoxide dismutase 3 (*SOD3*)) while upregulating hypoxia/glycolysis‐associated genes such as *PLOD2* and the glycolytic regulator 6‐phosphofructo‐2‐kinase/fructose‐2,6‐biphosphatase 3 (*PFKFB3*) (Figure S2B). Taken together, these patterns delineate SMC2 as a transitional/fibrotic state and SMC3 as an inflammation‐ and hypoxia‐biased state.

**FIGURE 2:**
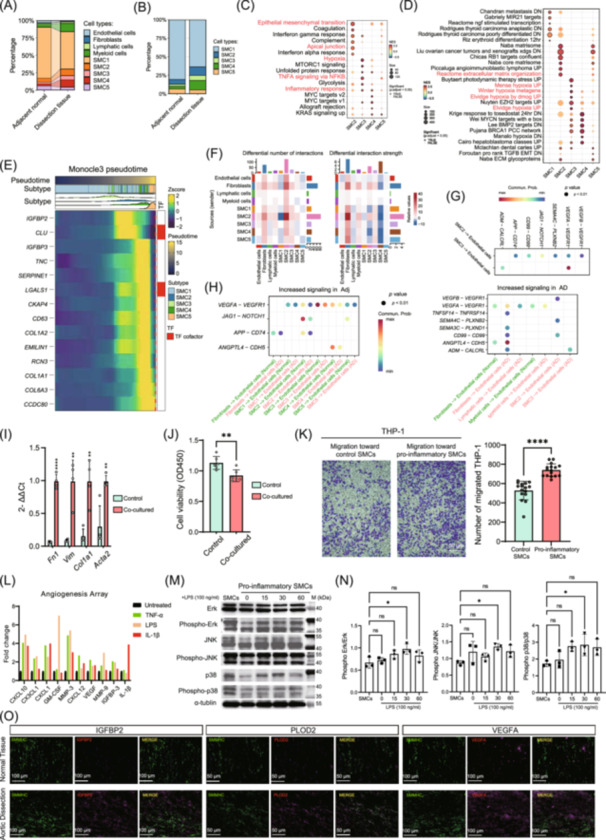
Identification and characterization of smooth muscle cell subpopulations in Aortic Dissection. (A) Comparative analysis of cell type distributions between 10 Aortic Dissection (AD) lesion libraries and 5 paired adjacent normal libraries. Mean percentages are calculated across all cells per group. (B) Comparison of smooth muscle cell (SMC) subtype proportions in 10 AD lesion libraries versus 5 paired adjacent normal libraries. (C and D) Gene set enrichment analysis (GSEA) results for (C) Hallmark pathways and (D) selected pathways, identifying distinct functional profiles of SMC subtypes (False Discovery Rate [FDR]‐adjusted *q* values). (E) Heatmap shows genes dynamically associated with pseudotime progression (*Monocle3*; Bonferroni‐adjusted *p* values). (F) Cell–cell communication analysis using *CellChat* showing differential number of inferred interactions (left) and interaction strength (right) between AD and adjacent normal tissues. (G) Differential signaling from SMC2 and SMC3 to endothelial cells via multiple pathways (CellChat‐derived *p* values). (H) Shift in signaling sources from SMCs to fibroblasts. In 5 paired adjacent normal libraries (left), SMCs are the predominant signaling sources, whereas fibroblasts contribute significantly to signaling in 10 AD lesion libraries (right) (*CellChat*‐derived *p* values). (I) Quantitative polymerase chain reaction (qPCR) results showing upregulation of fibrotic markers in endothelial cells co‐cultured with pro‐fibrotic‐SMCs (four biological replicates in each group, unpaired *t*‐test). (J) Bar plot quantifying the inhibition of endothelial cell proliferation when co‐cultured with pro‐fibrotic‐SMCs (six biological replicates in each group, unpaired *t*‐test). (K) Representative images and quantification of THP‐1 monocyte migration toward control SMCs or pro‐inflammatory SMCs in a Transwell migration assay (16 biological replicates in each group, unpaired *t*‐test). (L) Histogram depicting increased secretion of pro‐inflammatory factors by pro‐inflammatory‐SMCs upon stimulation with inflammatory mediators. (M, N) Western blot results indicating increased phosphorylation of extracellular signal‐regulated kinase (p‐ERK), c‐Jun N‐terminal kinase (JNK), and p38 mitogen‐activated protein kinase (p38) in pro‐inflammatory‐SMCs induction assays (*n* = 3 independent biological replicates per time point; one‐way analysis of variance [ANOVA] followed by Dunnett's multiple‐comparisons test vs. 0 h). (O) Immunofluorescence illustrating increased expression of insulin‐like growth factor binding protein 2 (*IGFBP2*), procollagen‐lysine 2‐oxoglutarate 5‐dioxygenase 2 (*PLOD2*), and vascular endothelial growth factor A (*VEGFA*) in dissection tissue compared to adjacent normal tissue. The data are presented as the means ± SD; *p < 0.05, **p < 0.01, ****p < 0.0001.

Pseudotime trajectory analysis identified critical genes driving SMC differentiation and phenotypic transitions (Figures 2E, S2C,D). The heatmap displays gene expression dynamics along the pseudotime trajectory, revealing distinct transcriptional programs associated with each SMC subtype. SMC1 demonstrated a relatively quiescent transcriptional profile, with the highest expression of contractile markers *MYH11* and *ACTA2* (Figure S2E), characterized by low expression of most trajectory‐associated genes except for baseline contractile markers, consistent with its maintenance of a differentiated contractile state with minimal transcriptional activation. Branch‐dependent gene dynamics suggested stepwise transitions along pseudotime. Along pseudotime, *CLU* increased with the emergence of the SMC2 state; in contrast, lectin galactoside‐binding soluble 1 (*LGALS1*) increased toward the SMC4‐associated state but was comparatively lower in the SMC5‐associated state, supporting clusterin (*CLU*) and *LGALS1* as branch‐associated marker genes for SMC state transitions (Figure S2F).

Beyond SMCs, we examined other cellular populations to understand the broader microenvironmental changes in AD. The relative proportions of endothelial cells (ECs) did not differ between AD and adjacent normal tissues, and subtype‐resolved GO analysis showed that EC1 was mainly enriched for cell‐migration pathways, whereas EC2 and EC3 were enriched for extracellular‐matrix and stromal‐like differentiation (Figure S2G–I).

Cell‐cell communication analysis revealed increased interaction strength in AD tissue, with inferred interactions rising from 659 to 897 and interaction strength from 33,788 to 34,862 (Figure S2J). Differential interaction heatmaps showed enhanced SMC and fibroblast signaling (Figure 2F). SMC2 and SMC3 displayed enhanced outgoing signaling toward ECs through the semaphorin 4C (*SEMA4C*)–plexin B2 (*PLXNB2*), jagged canonical notch ligand 1 (*JAG1*)–notch receptor 1 (*NOTCH1*), *VEGFA*–*VEGFR1*, and amyloid beta precursor protein (*APP*)–*CD74* axes (Figures 2G,H, S2K). Notably, *VEGFA*–*VEGFR1* signaling to ECs was redistributed, with SMCs predominating in Adj, whereas fibroblasts emerged as the major sender in AD (Figure 2H), with their detailed changes documented in Figure S2L,M. In vitro assays showed that *ANGPTL4* and *VEGFA* did not regulate each other's expression, and their co‐overexpression did not further enhance MAPK activation (Figure S3). Hypoxic stimulation induced a time‐dependent upregulation of *ANGPTL4* at both mRNA and protein levels in SMCs, with *VEGFA* showing a similar trend (Figure S4).

In vitro experiments validated these findings. Co‐culturing TGF‐β1‐induced SMC2‐like pro‐fibrotic‐SMCs cells with ECs (SVEC4‐10) induced endothelial‐to‐mesenchymal transition (EndMT), increasing fibrotic markers (fibronectin (*FN1*), vimentin *(VIM*), *COL1A1*, *ACTA2*) and reducing endothelial proliferation by 47% (Figure 2I,J). The primer sequences used are listed in Table S3. SMC3‐like pro‐inflammatory‐SMCs cells promoted THP‐1 monocyte migration (*p* < 0.0001) in a Transwell migration assay and secreted elevated inflammatory mediators (C‐X‐C motif chemokine ligand 10 (*CXCL10*), C‐X3‐C motif chemokine ligand 1 (*CX3CL1*), GM‐CSF) (Figure 2K,L). Western blot analysis confirmed MAPK pathway activation (phosphorylated extracellular signal‐regulated kinase (p‐ERK), c‐jun N‐terminal kinase (JNK), p38 mitogen‐activated protein kinase (p38)) in pro‐inflammatory‐SMCs cells (Figure 2M,N). Immunofluorescence verified elevated insulin‐like growth factor binding protein 2 (*IGFBP2*), *PLOD2*, and *VEGFA* in AD tissue, supporting roles in proliferation, ECM remodeling, and angiogenesis (Figure 2O). The *VEGFA* signaling shift aligns with its role in vascular pathology [14, 15], and the list of antibodies used in these experiments is provided in Table S4.

In summary, our scRNA‐seq analysis reveals that, beyond general phenotypic heterogeneity, specific SMC subsets exhibit distinct pathological programs in AD. We identify SMC2 as a pro‐fibrotic phenotype that promotes endothelial‐to‐mesenchymal transition via upregulation of collagen genes and ECM‐remodeling factors, while SMC3 displays a MAPK‐dependent synthetic‐inflammatory profile characterized by enhanced secretion of chemokines and recruitment of immune cells. Notably, thrombus‐positive AD samples demonstrate selective enrichment of SMC3 and upregulation of *ANGPTL4*, coinciding with an immunosuppressive M2 macrophage environment. These alterations are consistent with prior evidence linking inflammatory SMC phenotypic switching and macrophage polarization to matrix degradation, fibrosis, and vascular instability in AD [16, 17, 18]. Moreover, the observed shift in *VEGFA* signaling from SMCs to fibroblasts reflects disrupted vascular homeostasis, a mechanism implicated in pathological angiogenesis and increased vascular permeability [15, 19]. These findings define mechanistic links between SMC phenotype, inflammatory and fibrotic pathways, and thrombus status, thereby extending the current understanding of SMC‐driven pathology in AD.

This study has several limitations that warrant consideration. First, the use of adjacent “normal” aortic tissue as the control group, although lacking gross pathological changes, cannot fully exclude the presence of subclinical alterations, particularly in individuals with genetic predispositions or chronic cardiovascular risk factors. Second, our findings are based solely on transcriptomic observations without genotype stratification or adjustment. Such tissue may already harbor early molecular or structural changes, and, as all samples were collected at the time of surgery, they represent end‐stage disease. Consequently, some of the molecular signatures identified may reflect secondary responses to advanced tissue injury rather than primary pathogenic events, which may limit their immediate therapeutic relevance. Additionally, this work represents a preliminary screening of cell populations and gene expression changes, lacking functional validation of key genes, protein‐level confirmation, and in vivo experiments. Future studies incorporating truly healthy donor aortic tissue and integrating proteomics, functional assays, and animal models will be essential to validate these findings and identify early, causative therapeutic targets.

This study uncovers profound cellular and molecular alterations in AD, emphasizing the critical role of inflammatory SMCs and disrupted intercellular communication in disease progression. The distinct cellular signatures associated with thrombus formation highlight its importance as a pathological modifier in AD. These findings deepen our understanding of AD pathogenesis and propose potential therapeutic targets aimed at modulating SMC phenotypes to restore vascular integrity in AD patients.

## AUTHOR CONTRIBUTIONS

**Liang Shao**: Conceptualization; funding acquisition; writing—review and editing; investigation; methodology. **Fan Hu**: Investigation. **Ling‐Na Zhao**: Investigation. **Jian‐Ping Luo**: Formal analysis; writing—original draft. **Peng‐Tao Zou**: Writing—original draft; formal analysis. **Xiu Liu**: Formal analysis; writing—original draft. **Shao‐Yi Zheng**: Formal analysis; data curation; methodology; resources. **Cong Chen**: Formal analysis; writing—original draft. **Lin‐Xiong Ye**: Writing—original draft; formal analysis; data curation; methodology; software; visualization. **Yu‐Xuan Zhou**: Investigation; supervision. **Jiaqi Zhang**: Supervision. **Kaidi Jin**: Supervision. **Ping Zhang**: Conceptualization; funding acquisition; investigation; methodology; writing—review and editing. All authors have read the final manuscript and approved it for publication.

## CONFLICT OF INTEREST STATEMENT

The authors declare no conflicts of interest.

## ETHICS STATEMENT

All participants provided written informed consent, and the research has been approved by the Institutional Ethics Committees of Nanfang Hospital of Southern Medical University (No. NFEC‐2023‐447).

## Supporting information


**Figure S1.** Supplementary marker, clinical association, and myeloid enrichment analyses.
**Figure S2.** Extended analyses of cell programs and intercellular signaling.
**Figure S3.** Effects of *VEGFA* and *ANGPTL4* overexpression on reciprocal expression and MAPK pathway activation in smooth muscle cells.
**Figure S4.** Hypoxia induces time‐dependent upregulation of *ANGPTL4* and *VEGFA* in smooth muscle cells.

**Table S1.** Clinical characteristics of the overall study cohort.
**Table S2.** scRNA‐seq sample manifest and key clinical annotations (*n* = 15 libraries).
**Table S3.** Primer sequences for gene amplification.
**Table S4.** Antibodies used.

## Data Availability

The data that support the findings of this study are available from the corresponding author upon reasonable request. The scRNA‐seq data reported in this study are not currently publicly available due to institutional, ethical, and legal restrictions on data sharing and the involvement of human participant‐related information. The data are available from the corresponding author upon reasonable request and subject to local rules and regulations. The processed data and analysis scripts used are available on GitHub (https://github.com/yelinxiong/imeta_AD). Supplementary materials (methods, figures, tables, graphical abstract, slides, videos, Chinese translated version, and update materials) may be found in the online DOI or iMeta Science http://www.imeta.science/.

## References

[1] Zhu, Yuanjia , Bharathi Lingala , Michael Baiocchi , Jacqueline J. Tao , Veronica Toro Arana , Jason W. Khoo , Kiah M. Williams , et al. 2020. “Type A Aortic Dissection—Experience Over 5 Decades.” Journal of the American College of Cardiology 76: 1703–1713. 10.1016/j.jacc.2020.07.061 33004136

[2] Silaschi, Miriam , Jonathan Byrne , and Olaf Wendler . 2017. “Aortic Dissection: Medical, Interventional and Surgical Management.” Heart 103: 78–87. 10.1136/heartjnl-2015-308284 27733536

[3] Tadros, Rami O. , Gilbert H. L. Tang , Hanna J. Barnes , Idine Mousavi , Jason C. Kovacic , Peter Faries , Jeffrey W. Olin , Michael L. Marin , and David H. Adams . 2019. “Optimal Treatment of Uncomplicated Type B Aortic Dissection.” Journal of the American College of Cardiology 74: 1494–1504. 10.1016/j.jacc.2019.07.063 31514953

[4] Okita, Yutaka . 2023. “Aortic Dissection is More Violent in the Young.” European Journal of Cardio‐Thoracic Surgery 63: ezad209. 10.1093/ejcts/ezad209 37233032

[5] Gawinecka, Joanna , Felix Schönrath , and Arnold von Eckardstein . 2017. “Acute Aortic Dissection: Pathogenesis, Risk Factors and Diagnosis.” Swiss Medical Weekly 147: w14489. 10.4414/smw.2017.14489 28871571

[6] Yin, Zhi‐Qiang , Hua Han , Xianchun Yan , and Qi‐Jun Zheng . 2023. “Research Progress on the Pathogenesis of Aortic Dissection.” Current Problems in Cardiology 48: 101249. 10.1016/j.cpcardiol.2022.101249 35568084

[7] Chen, Yinan , Tao Zhang , Fang Yao , Xiang Gao , Dandan Li , Shufang Fu , Lin Mao , et al. 2022. “Dysregulation of Interaction Between Loxhigh Fibroblast and Smooth Muscle Cells Contributes to the Pathogenesis of Aortic Dissection.” Theranostics 12: 910–928. 10.7150/thno.66059 34976220 PMC8692905

[8] Zhou, Yihong , Tingyu Wang , Hongyou Fan , Shan Liu , Xiaomei Teng , Lianbo Shao , and Zhenya Shen . 2024. “Research Progress on the Pathogenesis of Aortic Aneurysm and Dissection in Metabolism.” Current Problems in Cardiology 49: 102040. 10.1016/j.cpcardiol.2023.102040 37595858

[9] Zhang, Li , Zhihuang Qiu , Hui Zheng , Xi Yang , Jianqiang Ye , Jian He , Yumei Li , et al. 2022. “Single Cell RNA Sequencing Reveals the Pathogenesis of Aortic Dissection Caused by Hypertension and Marfan Syndrome.” Frontiers in Cell and Developmental Biology 10: 880320. 10.3389/fcell.2022.880320 35800890 PMC9253298

[10] Liu, Xuanyu , Wen Chen , Guoyan Zhu , Hang Yang , Wenke Li , Mingyao Luo , Chang Shu , and Zhou Zhou . 2022. “Single‐Cell RNA Sequencing Identifies an Il1rn+/Trem1+ Macrophage Subpopulation as a Cellular Target for Mitigating the Progression of Thoracic Aortic Aneurysm and Dissection.” Cell Discovery 8: 11. 10.1038/s41421-021-00362-2 35132073 PMC8821555

[11] Zhang, Bin , Kuan Zeng , Rui‐Cong Guan , Hui‐Qi Jiang , Yong‐Jia Qiang , Qing Zhang , Mo Yang , Bao‐Ping Deng , and Yan‐Qi Yang . 2023. “Single‐Cell RNA‐seq Analysis Reveals Macrophages are Involved in the Pathogenesis of Human Sporadic Acute Type A Aortic Dissection.” Biomolecules 13: 399. 10.3390/biom13020399 36830768 PMC9952989

[12] Im Cho, Dong , Hye‐jin Kang , Ju Hee Jeon , Gwang Hyeon Eom , Hyang Hee Cho , Mi Ra Kim , Meeyoung Cho , et al. 2019. “Antiinflammatory Activity of ANGPTL4 Facilitates Macrophage Polarization to Induce Cardiac Repair.” JCI Insight 4: e125437. 10.1172/jci.insight.125437 31434807 PMC6777833

[13] Huang, Weifeng , Liqing Jiang , Yingsong Jiang , Shanshan Li , Wanqi Liu , Kezhen Zong , Dadi Peng , Zhongjun Wu , and Zuotian Huang . 2025. “ANGPTL4 Induces Kupffer Cell M2 Polarization to Mitigate Acute Rejection in Liver Transplantation.” Scientific Reports 15: 986. 10.1038/s41598-024-81832-x 39762255 PMC11704181

[14] Sjöberg, Elin , Marit Melssen , Mark Richards , Yindi Ding , Catarina Chanoca , Dongying Chen , Emmanuel Nwadozi , et al. 2023. “Endothelial VEGFR2‐PLCγ Signaling Regulates Vascular Permeability and Antitumor Immunity Through eNOS/Src.” The Journal of Clinical Investigation 133: e161366. 10.1172/JCI161366 37651195 PMC10575733

[15] Lutter, Sophie , Sherry Xie , Florence Tatin , and Taija Makinen . 2012. “Smooth Muscle–Endothelial Cell Communication Activates Reelin Signaling and Regulates Lymphatic Vessel Formation.” Journal of Cell Biology 197: 837–849. 10.1083/jcb.201110132 22665518 PMC3373399

[16] Chakraborty, Abhijit , Yanming Li , Chen Zhang , Yang Li , Kimberly R. Rebello , Shengyu Li , Samantha Xu , et al. 2023. “Epigenetic Induction of Smooth Muscle Cell Phenotypic Alterations in Aortic Aneurysms and Dissections.” Circulation 148: 959–977. 10.1161/CIRCULATIONAHA.123.063332 37555319 PMC10529114

[17] Wang, Juan , Qiang Wu , Xinyu Wang , Hongbin Liu , Mulei Chen , Li Xu , Ze Zhang , et al. 2024. “Targeting Macrophage Phenotypes and Metabolism as Novel Therapeutic Approaches in Atherosclerosis and Related Cardiovascular Diseases.” Current Atherosclerosis Reports 26: 573–588. 10.1007/s11883-024-01229-z 39133247 PMC11392985

[18] Francis, Gordon A . 2023. “The Greatly Under‐Represented Role of Smooth Muscle Cells in Atherosclerosis.” Current Atherosclerosis Reports 25: 741–749. 10.1007/s11883-023-01145-8 37665492 PMC10564813

[19] Ding, Xili , Qin An , Weikang Zhao , Yang Song , Xiaokai Tang , Jing Wang , Chih‐Chiang Chang , et al. 2022. “Distinct Patterns of Responses in Endothelial Cells and Smooth Muscle Cells Following Vascular Injury.” JCI Insight 7: e153769. 10.1172/jci.insight.153769 36278486 PMC9714785

