# Supplementary material for: Single‐cell transcriptomics reveals cellular heterogeneity and phenotypic transitions of smooth muscle cells in aortic dissection
Source: Imeta. 2026 Apr 21;5(2):e70124. doi: 10.1002/imt2.70124 (PMC13147948; doi:10.1002/imt2.70124)
Supplement: Supplementary file 1 — Figure S1. Supplementary marker, clinical association, and myeloid enrichment analyses. Figure S2. Extended analyses of cell programs and intercellular signaling. Figure S3. Effects of VEGFA and ANGPTL4 overexpression on reciprocal expression and MAPK pathway activation in smooth muscle cells. Figure S4. Hypoxia induces time‐dependent upregulation of ANGPTL4 and VEGFA in smooth muscle cells. [file IMT2-5-e70124-s001.docx]

**Supporting information to**

**Single-cell transcriptomics reveals cellular heterogeneity and phenotypic transitions of smooth muscle cells in aortic dissection**

**Running title:** Heterogeneity of smooth muscle cells in aortic dissection

Liang Shao^1,2,3*#^, Fan Hu^4#^, Ling-Na Zhao^5#^, Jian-Ping Luo^6^, Peng-Tao Zou^3^, Xiu Liu^5^,

Shao-Yi Zheng^5^, Cong Chen^7^, Lin-Xiong Ye ^8^, Yu-Xuan Zhou^3^, Jiaqi Zhang^8^, Kaidi Jin^9^, Ping Zhang^4^^*^

^1^Department of Cardiology, the Second Affiliated Hospital, University of South China, Hengyang 421001, China

^2^Hengyang Medical School, University of South China, Hengyang 421001, China

^3^Department of Cardiology, Jiangxi Provincial People's Hospital, The First Affiliated Hospital of Nanchang Medical College, Nanchang 330013, China

^4^Department of Neurology, Jiangxi Provincial People's Hospital, The First Affiliated Hospital of Nanchang Medical College, Nanchang 330013, China

^5^Department of Cardiovascular Surgery, Nanfang Hospital, Southern Medical University, Guangzhou 510515, China

^6^Department of Cardiology, Ganzhou People's Hospital, Ganzhou 314000, China

^7^Department of General Surgery, Pancreatic Disease Center, Ruijin Hospital, Shanghai Jiao Tong University School of Medicine, Shanghai 200001, China

^8^Department of Biological Medicines & Shanghai Engineering Research Center of Immunotherapeutics, School of Pharmacy, Fudan University, Shanghai 200001, China

^9^Department of Forensic Medicine, School of Basic Medical Sciences, Fudan University, Shanghai 200001, China

^#^These authors contributed equally: Liang Shao, Fan Hu, Ling-Na Zhao.

*Correspondence:

shaoliang021224@hotmail.com (Liang Shao); zhangkiki520@163.com (Ping Zhang)

**Materials and methods**

**Patient cohort and sample collection**

After general anesthesia, patients undergoing surgical repair for acute aortic disease (including Stanford type A aortic dissection and intramural hematoma) were approached via median sternotomy. The ascending aortic dissection with extended tear was found. Femoral artery-venous extracorporeal circulation was performed immediately. Multiple lesions were found in the aortic intima wall. The lesioned part of the aorta was removed, the original aorta was embedded and sutured with artificial blood vessels, the aortic root was repeatedly vented, the aorta was opened, the chest cavity was closed for drainage, the sternum was closed, and the incision was sutured. After the operation, the vascular tissue of the lesioned part of the aorta and the normal vascular tissue next to the lesion were collected for sampling. The diagnosis was confirmed by computed tomographic angiography (CTA) [1]. Immediately after excision, specimens were immersed in ice-cold transport buffer, maintained at 4 ℃ on ice, and transported to the laboratory. In parallel, a small portion of each specimen was snap-frozen in liquid nitrogen and stored at −80 ℃ for contingency analyses. All patients were recruited from the Department of Cardiovascular Surgery, Nanfang Hospital, Southern Medical University. The study was approved by the ethics committee of Southern Medical University and conformed to the principles of the Declaration of Helsinki [2]. Informed consent was obtained from each patient. Inclusion criteria were adults undergoing surgical repair for aortic dissection (Stanford type A aortic dissection or intramural hematoma) with diagnosis confirmed by CTA; exclusion criteria were iatrogenic/traumatic/infectious aortitis, prior surgery at the sampled aortic segment, clinically syndromic presentations, active infectious disease, renal failure, or major surgery/severe trauma within the preceding three months. Clinical characteristics of the overall study cohort are summarized in Table S1.

**Single cell RNA-seq (scRNA-seq) sequencing experiments**

ScRNA-seq was performed on diseased aortic tissue and adjacent aortic tissue collected during surgery. The adjacent normal libraries were derived from aortic tissue collected adjacent to the lesion from the same surgical patients, rather than from unrelated healthy donors. A complete library-level sample manifest (n = 15), including tissue type, thrombus annotation, and patient mapping for each scRNA-seq library, is provided in Table S2. Cells were prepared at a concentration of 1,000 cells per μl and processed using the 10X Chromium Single Cell Platform (10X Genomics) with the Chromium Next GEM Single Cell 3ʹ Kit v3.1 [3] (10X Genomics; PN-1000268), following the manufacturer's guidelines. The workflow included the generation of gel beads in emulsion (GEMs), barcoding, GEM-RT cleanup, complementary DNA (cDNA) amplification, and library preparation, all conducted as per the provided protocol. Each library was generated from a single patient sample (no pooling), and no cell hashing was performed. Library quantification was performed using the Qubit 4 Fluorometer (Invitrogen; Q33238) prior to pooling. Sequencing of the final library pool was carried out on the Illumina Nova6000 system with 150-base-pair paired-end reads. Initial data processing was conducted using the Cell Ranger pipeline (10× Genomics), which was employed to preprocess raw sequencing data. Filtered feature-barcode matrices were obtained and used for downstream analyses.

**Data processing and quality control**

All data analyses were conducted in the R environment (version 4.2.3) using the *Seurat* package [4] (version 4.4.0). The filtered feature-barcode matrices were imported into R using *Seurat*. Initial quality control (QC) metrics were visualized with the *PrePlots* function. Cells were filtered based on gene detection levels, unique molecular identifier (UMI) counts, and the percentage of mitochondrial gene expression (percent.mt < 10%). Filtering effects were visualized using the *FilterPlots* function. Doublet cells were identified and removed using the *scDblFinder* method. Because libraries were generated per patient and not pooled, demultiplexing by hashing was not required.

**Normalization, feature selection, and dimensionality reduction**

Following QC and filtering, the data were normalized using the *NormalizeData* function. The *FindVariableFeatures* function was applied to identify 2,000 highly variable features. Data scaling was conducted using the *ScaleData* function, and dimensionality reduction was performed using principal component analysis (PCA) via the *RunPCA* function. The first 20 principal components (PCs) were selected for further analysis. To correct for batch effects across samples, the Harmony algorithm was applied with sample as the batch variable.

**Clustering and cell type annotation**

For clustering and visualization, uniform manifold approximation and projection (UMAP) was applied using the *RunUMAP* function. Cells were clustered by first generating a neighbor graph using *FindNeighbors*, followed by clustering with the *FindClusters* functions at a resolution of 0.2. Clusters were annotated by identifying marker genes and using reference-based annotation via the *SingleR* package [5], with the Human Primary Cell Atlas as the reference dataset.

**Differential expression analysis**

Differential gene expression analysis between AD and adjacent normal tissue was performed using the *FindMarkers* function in *Seurat*. The Wilcoxon rank-sum test was employed to identify genes differentially expressed within each cell type, comparing AD tissue and normal tissue. For thrombus-related differential expression analysis, the Model-based Analysis of Single-cell Transcriptomics (MAST) test was used with SMC subtype as a latent variable to control for compositional differences. Genes were considered significantly differentially expressed if the adjusted *p* value was < 0.05 and the | log_2_ (fold change) | exceeded 0.5.

**Gene set enrichment analysis**

Gene set enrichment analysis (GSEA) was conducted using the *clusterProfiler* package [6]. For each comparison, ranked gene lists based on log_2_ fold changes were generated. Gene sets from Molecular Signatures Database (MSigDB) v7.2, including the Hallmark, C2 (curated), and C5 (GO) collections, were used in the analysis. GSEA was run with a minimum gene set size of 5 and a maximum of 500. Pathways with an adjusted *p* value of < 0.05 were deemed significantly enriched.

**Cell-cell communication analysis**

Cell-cell communication analyses were performed using the *CellChat* package [7], separately for AD and adjacent normal tissue samples. Significant interactions were identified with a *p* value threshold of 0.05. Differential cell-cell communication between AD and normal samples was assessed using the *compareInteractions* and *netAnalysis_signalingChanges_scatter* functions in *CellChat*.

**Trajectory analysis**

Trajectory analysis was conducted using the *Monocle3* package [8]. The *Seurat* object was converted to a *cell_data_set* object, and UMAP coordinates from *Seurat* were employed for visualization. The trajectory graph was constructed utilizing the *learn_graph* function, while pseudotime was calculated using the *order_cells* function. We set the pseudotime origin to the contractile SMC cluster (SMC1), defined by high expression of canonical markers (myosin heavy chain 11 (*MYH11*), actin alpha 2 (*ACTA2*)).

**Data visualization**

Various visualization techniques were applied throughout the analysis to facilitate data interpretation. UMAP plots, generated using *Seurat*'s *DimPlot* and *FeaturePlot* functions, were utilized to depict cell clusters and gene expression patterns. Heatmaps illustrating marker gene expression across cell types were created using the *DoHeatmap* function. Violin plots, which allowed for comparing gene expression across different conditions, were generated by the *VlnPlot* function. Additionally, dot plots showing gene expression levels and the percentage of cells expressing each gene were produced using a custom *mDotplot* function. Bar plots depicting cell type proportions across conditions were generated using the *dittoBarPlot* function from the *dittoSeq* package. Volcano plots displaying differential expression results were created with the *EnhancedVolcano* package [9].

**Induction of SMC phenotypes​**

HASMC (Human Aortic Smooth Muscle Cell) were cultured in complete growth medium (Dulbecco's Modified Eagle Medium (DMEM) supplemented with 10% fetal bovine serum (FBS), 100 U/mL penicillin, and 100 µg/mL streptomycin). To induce a pro-fibrotic phenotype (SMC2-like), the culture medium was replaced with serum-free medium containing 5 ng/mL recombinant human TGF-β1. Cells were incubated for 48 h to establish the phenotype. To induce a pro-inflammatory phenotype (SMC3-like), HASMCs were stimulated with 100 ng/mL Lipopolysaccharide (LPS) in serum-free medium for 24 h.

**Hypoxia stimulation**

SMCs were exposed to hypoxic conditions using a sealed anaerobic culture system (Mitsubishi Gas Chemical, Japan; 2.5 L anaerobic culture jar, Cat. No. C-31). Cells were incubated under hypoxia for 6 h, 12 h, and 24 h, with normoxic cultures serving as controls. Cells were collected at each time point for total RNA extraction and quantitative reverse transcription polymerase chain reaction (qPCR), or for protein extraction and Western blot analysis.

**In vitro overexpression and co-overexpression of ANGPTL4 and VEGFA**

To investigate potential regulatory interactions between ANGPTL4 and VEGFA SMC, cells were transfected with plasmids encoding ANGPTL4, VEGFA, or both genes simultaneously. Briefly, SMCs were seeded in 6-well plates and transfected at ~70–80% confluence using Lipofectamine 2000 (11668027, Thermo Fisher Scientific) according to the manufacturer's instructions. Empty vector was used as a negative control. At 24–48 h post-transfection, cells were harvested for downstream analyses.

**Cell Counting Kit-8 (CCK8) assays**

Cell proliferation was assessed using the CCK8 assay (Beyotime Biotechnology, C0038). After 24 h of co-culture with SMC or pro-fibrotic-SMCs cells, the CCK8 solution was added to the culture medium of SVEC4-10 cells in 24-well plates. Following a 60-minute incubation at 37°C, the supernatant was collected, and absorbance was measured at 450 nm.

**QPCR experiments**

Total RNA was extracted using Trizol (Sigma) according to the manufacturer's protocol. cDNA synthesis was performed using Hifair® II 1st Strand cDNA Synthesis SuperMix (Yeason, 11120ES60) [10]. PCR reactions were conducted using QK Platinum SYBR Green Master Mix (Thermo Scientific, A57155) on a LightCycler® 480 Instrument II (Roche). The primers used are listed in Table S3.

**Cell migration assays**

THP-1 cells were seeded onto Transwell® permeable inserts (Corning, 6.5 mm diameter, 5 μm pore size), which were placed in 24-well plates. SMC or pro-inflammatory-SMCs cells were seeded separately in 24-well culture plates. On the following day, the media were replaced with serum-free media, and the inserts containing THP-1 cells were transferred onto the wells with SMC or pro-inflammatory-SMCs cells. After 24 h of co-culture, the membranes were stained with 0.1% crystal violet for 5 minutes, washed with PBS, and the undersides of the membrane were examined under a light microscope.

**Proteome profiler mouse angiogenesis array**

The supernatant from SMCs stimulated with LPS, tumor necrosis factor alpha (TNF-α), or interleukin-1 beta (IL-1β) for 24 h was collected and analyzed using the Proteome Profiler Mouse Angiogenesis Array Kit (Bio-Techne, ARY015), following the manufacturer's instructions.

**Western blot experiments**

Cells for Western blot were harvested using trypsin and washed twice with PBS. The cells were lysed using radioimmunoprecipitation assay (RIPA) buffer (Beyotime Biotechnology, P0013B) containing protease (P1005) and phosphatase inhibitors (P1081). Lysates were incubated on ice for 30 minutes with vortexing every 10 minutes, then centrifuged at 12,000 rpm for 30 minutes at 4°C. The soluble protein fraction was collected for Western blot analysis [11]. The following antibodies used are listed in Table S4.

**Tyramide signal amplification**

Tyramide signal amplification (TSA) was performed on tissue sections, beginning with deparaffinization and rehydration of paraffin-embedded sections or thawing of frozen sections. Optional antigen retrieval was conducted using heat-induced methods. Nonspecific binding sites were blocked using serum or bovine serum albumin (BSA). The sections were incubated with primary antibodies, followed by horseradish peroxidase (HRP)-conjugated secondary antibodies. After washing, TSA reagent (tyramide conjugated to a fluorophore or biotin) was applied, where HRP catalyzed the deposition of tyramide [12]. For fluorescence detection, sections were counterstained with 4′,6-diamidino-2-phenylindole (DAPI) and visualized. For chromogenic detection, streptavidin-HRP and a substrate like 3,3′-diaminobenzidine (DAB) were used before examination under microscopy. The antibodies utilized are listed in Table S4.

**Statistical analysis**

Statistical analyses were conducted using GraphPad Prism software (version 8.0; GraphPad Software, San Diego, CA, USA) [13]. An unpaired t-test was applied to compare differences between two groups with normally distributed data, while one-way ANOVA was used for comparisons among multiple groups with normally distributed data. For non-normally distributed data, the Mann–Whitney U test was used for two-group comparisons, and the Kruskal–Wallis test followed by Dunn's multiple comparisons test was used for multi-group comparisons. Data are presented as mean ± standard deviation (SD). Statistical significance was defined as *p* < 0.05. All statistical methods used are detailed in the respective figure legends.

**REFERENCE**

1. Erbel, Raimund, Victor Aboyans, Catherine Boileau, Eduardo Bossone, Roberto Di Bartolomeo, Holger Eggebrecht, Arturo Evangelista, et al. 2014. “2014 ESC guidelines on the diagnosis and treatment of aortic diseases.” *Polish Heart Journal (Kardiologia Polska)* 72: 1169–1252. <https://doi.org/10.1093/eurheartj/ehu281>
2. Association, World Medical. 2013. “World Medical Association Declaration of Helsinki: ethical principles for medical research involving human subjects.” *JAMA* 310: 2191–2194. <https://doi.org/10.1001/jama.2013.281053>
3. Zheng, Grace XY, Jessica M Terry, Phillip Belgrader, Paul Ryvkin, Zachary W Bent, Ryan Wilson, Solongo B Ziraldo, et al. 2017. “Massively parallel digital transcriptional profiling of single cells.” *Nature Communications* 8: 14049. <https://doi.org/10.1038/ncomms14049>
4. Stuart, Tim, Andrew Butler, Paul Hoffman, Christoph Hafemeister, Efthymia Papalexi, William M Mauck, Yuhan Hao, et al. 2019. “Comprehensive integration of single-cell data.” *Cell* 177: 1888–1902. e1821. <https://doi.org/10.1016/j.cell.2019.05.031>
5. Aran, Dvir, Agnieszka P Looney, Leqian Liu, Esther Wu, Valerie Fong, Austin Hsu, Suzanna Chak, et al. 2019. “Reference-based analysis of lung single-cell sequencing reveals a transitional profibrotic macrophage.” *Nature Immunology* 20: 163–172. https://doi.org/10.1038/s41590-018-0276-y
6. Yu, Guangchuang, Li-Gen Wang, Yanyan Han, Qing-Yu He. 2012. “clusterProfiler: an R package for comparing biological themes among gene clusters.” *Omics: a Journal of Integrative Biology* 16: 284–287. <https://doi.org/10.1089/omi.2011.0118>
7. Jin, Suoqin, Christian F Guerrero-Juarez, Lihua Zhang, Ivan Chang, Raul Ramos, Chen-Hsiang Kuan, Peggy Myung, et al. 2021. “Inference and analysis of cell-cell communication using CellChat.” *Nature Communications* 12: 1088. https://doi.org/10.1038/s41467-021-21246-9
8. Cao, Junyue, Malte Spielmann, Xiaojie Qiu, Xingfan Huang, Daniel M Ibrahim, Andrew J Hill, Fan Zhang, et al. 2019. “The single-cell transcriptional landscape of mammalian organogenesis.” *Nature* 566: 496–502. https://doi.org/10.1038/s41586-019-0969-x
9. Blighe, Kevin, Sharmila Rana, and Myles Lewis. 2023. EnhancedVolcano: Publication-Ready Volcano Plots with Enhanced Colouring and Labeling. R package. https://doi.org/10.18129/B9.bioc.EnhancedVolcano
10. Bustin, Stephen A, Vladimir Benes, Jeremy A Garson, Jan Hellemans, Jim Huggett, Mikael Kubista, Reinhold Mueller, Tania Nolan, et al. 2009. The MIQE Guidelines: M inimum I nformation for Publication of Q uantitative Real-Time PCR E xperiments. 2009. https://doi.org/10.1373/clinchem.2008.112797
11. Mahmood, Tahrin, Ping-Chang Yang. 2012. “Western blot: technique, theory, and trouble shooting.” *North American Journal of Medical Sciences* 4: 429. <https://doi.org/10.4103/1947-2714.100998>
12. Bobrow, Mark N, Thomas D Harris, Krista J Shaughnessy, Gerald J Litt. 1989. “Catalyzed reporter deposition, a novel method of signal amplification application to immunoassays.” *Journal of Immunological Methods* 125: 279–285. <https://doi.org/10.1016/0022-1759(89)90104-x>
13. Kim, Jonghae, Dong Hyuck Kim, Sang Gyu Kwak. 2024. “Comprehensive guidelines for appropriate statistical analysis methods in research.” *Korean Journal of Anesthesiology* 77: 503–517. <https://doi.org/10.4097/kja.24016>

**Supplementary figures**

**
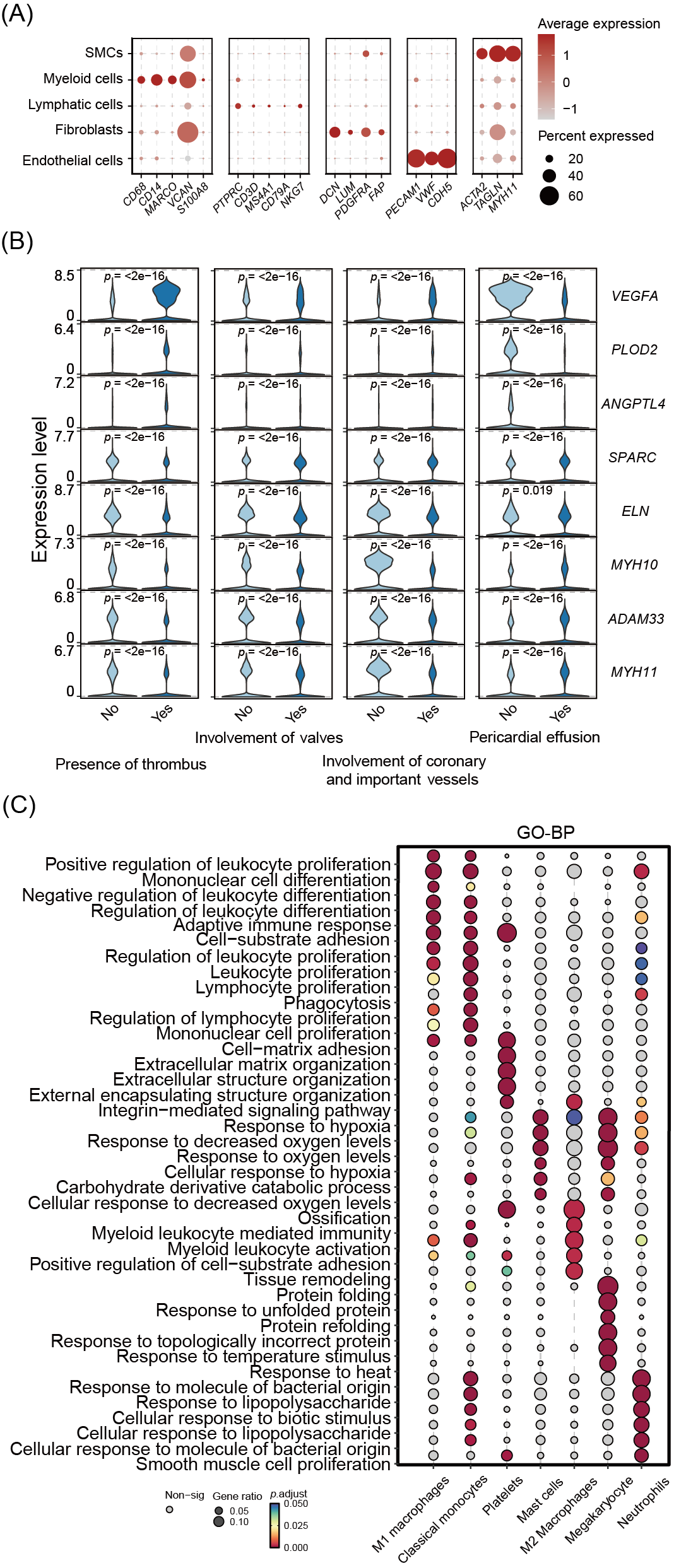
**

**Figure S1 Supplementary marker, clinical association, and myeloid enrichment analyses.** (A) Canonical markers used for major cell-type annotation. (B) Expression of representative thrombus-associated genes in SMCs stratified by thrombus status and clinical severity features (Wilcoxon rank-sum test). (C) Gene Ontology Biological Process (GO-BP) enrichment across myeloid subtypes (False Discovery Rate (FDR)-adjusted *q* values).

**
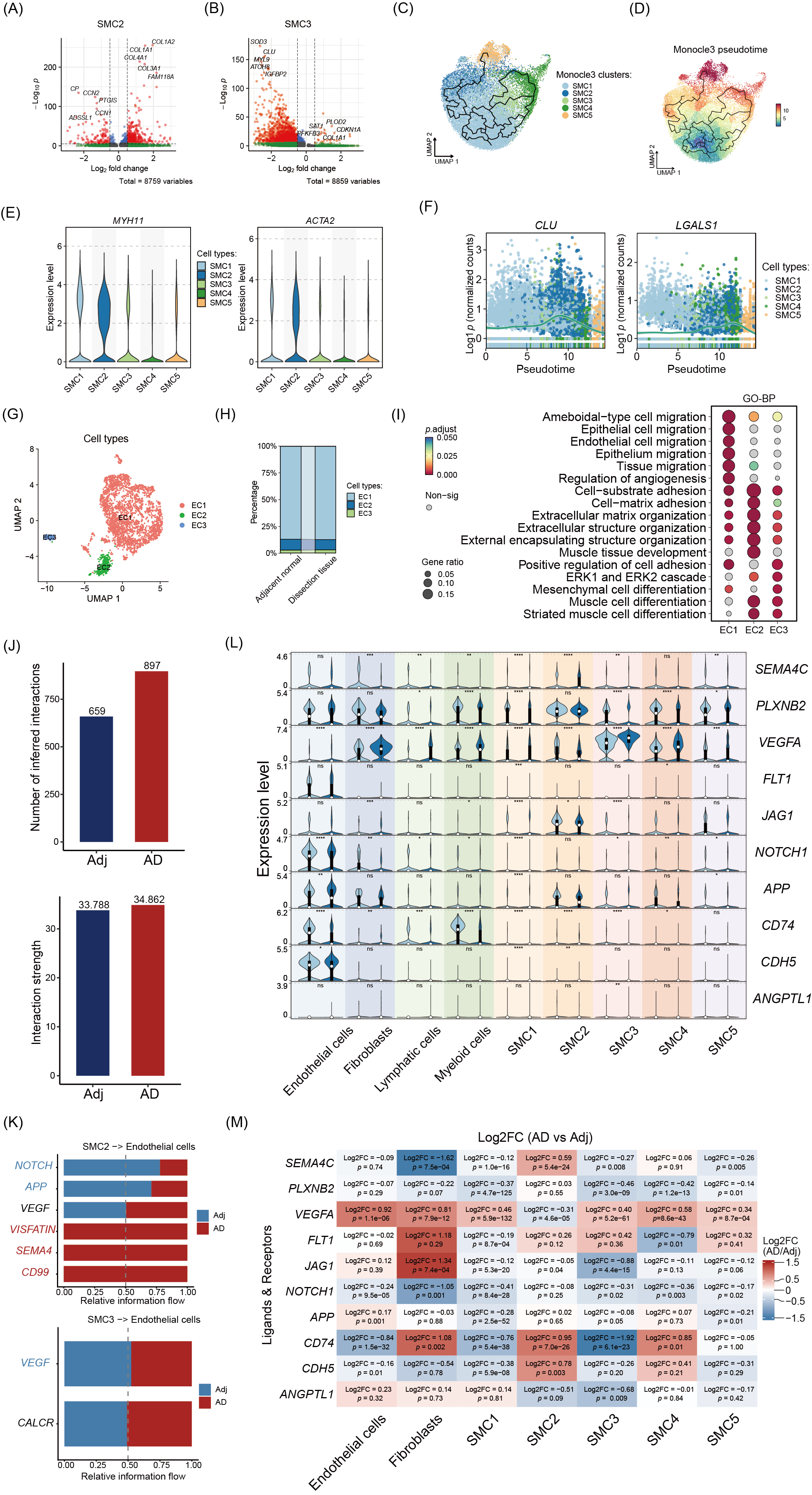
**

**Figure S2** **Extended analyses of cell programs and intercellular signaling.** (A and B) Volcano plots of differentially expressed genes in smooth muscle cell subtype 2 (SMC2) (A) and smooth muscle cell subtype 3 (SMC3) (B) comparing aortic dissection (AD) versus adjacent normal (Adj) tissue (two-sided Wilcoxon test; adjusted *p* values). (C) Uniform Manifold Approximation and Projection (UMAP) of smooth muscle cells (SMCs) with Monocle3 trajectory backbones overlaid; clusters correspond to SMC1–SMC5. (D) Pseudotime map of SMCs. (E) Violin plots of myosin heavy chain 11 (*MYH11*) and actin alpha 2 (*ACTA2*) across SMC subtypes. (F) Branch-associated expression dynamics of clusterin (*CLU*) and lectin galactoside-binding soluble 1 (*LGALS1*) along pseudotime. (G) UMAP of endothelial cells (ECs). (H) endothelial cell (EC) subtype proportions. (I) Gene Ontology Biological Process (GO-BP) enrichment across EC subtypes (False Discovery Rate [FDR]-adjusted *q* values). (J) *CellChat* summary statistics showing the number of inferred interactions (top) and total interaction strength (bottom) in AD versus Adj. (K) Relative information flow from SMC2/SMC3 to endothelial cells in AD versus Adj. (L) Violin plots of selected ligand/receptor genes across major cell types (Wilcoxon rank-sum test). (M) Heatmap summarizing ligand/receptor expression changes (log_2_ fold change (log_2_FC), AD vs Adj) with corresponding *p* values (Wilcoxon rank-sum test). For all statistical analyses, significance thresholds were set as follows: **p* < 0.05; ***p* < 0.01; ****p* < 0.001; *****p* < 0.0001.


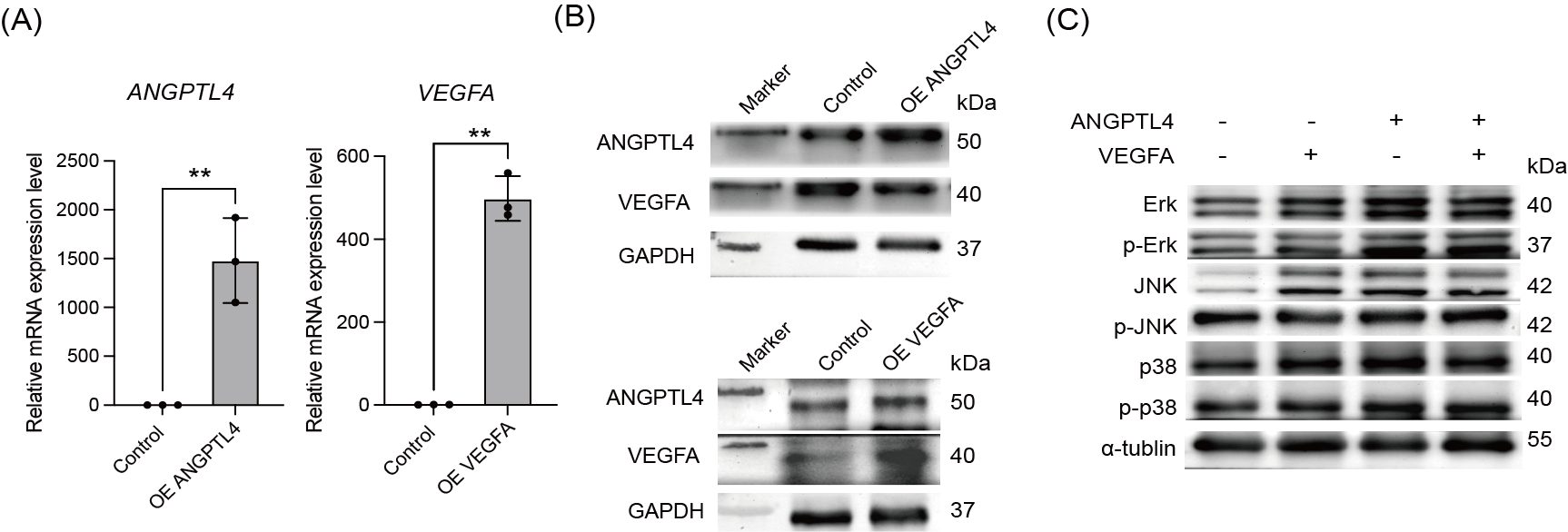


**Figure S3 Effects of *VEGFA* and *ANGPTL4* overexpression on reciprocal expression and mitogen-activated protein kinase (MAPK) pathway activation in SMCs.** (A) Quantitative reverse transcription polymerase chain reaction (qPCR) validation of angiopoietin-like 4 (*ANGPTL4*) and vascular endothelial growth factor A (*VEGFA*) overexpression efficiency in smooth muscle cells (SMCs) (n = 3). (B) Western blot analysis showing that overexpression of ANGPTL4 or VEGFA does not significantly alter the expression level of the other gene in SMCs (n = 3). (C) Western blot analysis of mitogen-activated protein kinase (MAPK) pathway activation (extracellular signal-regulated kinase [ERK], c-Jun N-terminal kinase [JNK], and p38 MAPK) in SMCs following ANGPTL4 overexpression, VEGFA overexpression, or co-overexpression of both genes. Statistical analysis was performed using a two-tailed unpaired Student's t-test. The data are presented as the means ± SD; * *p* < 0.05, ** *p* < 0.01.


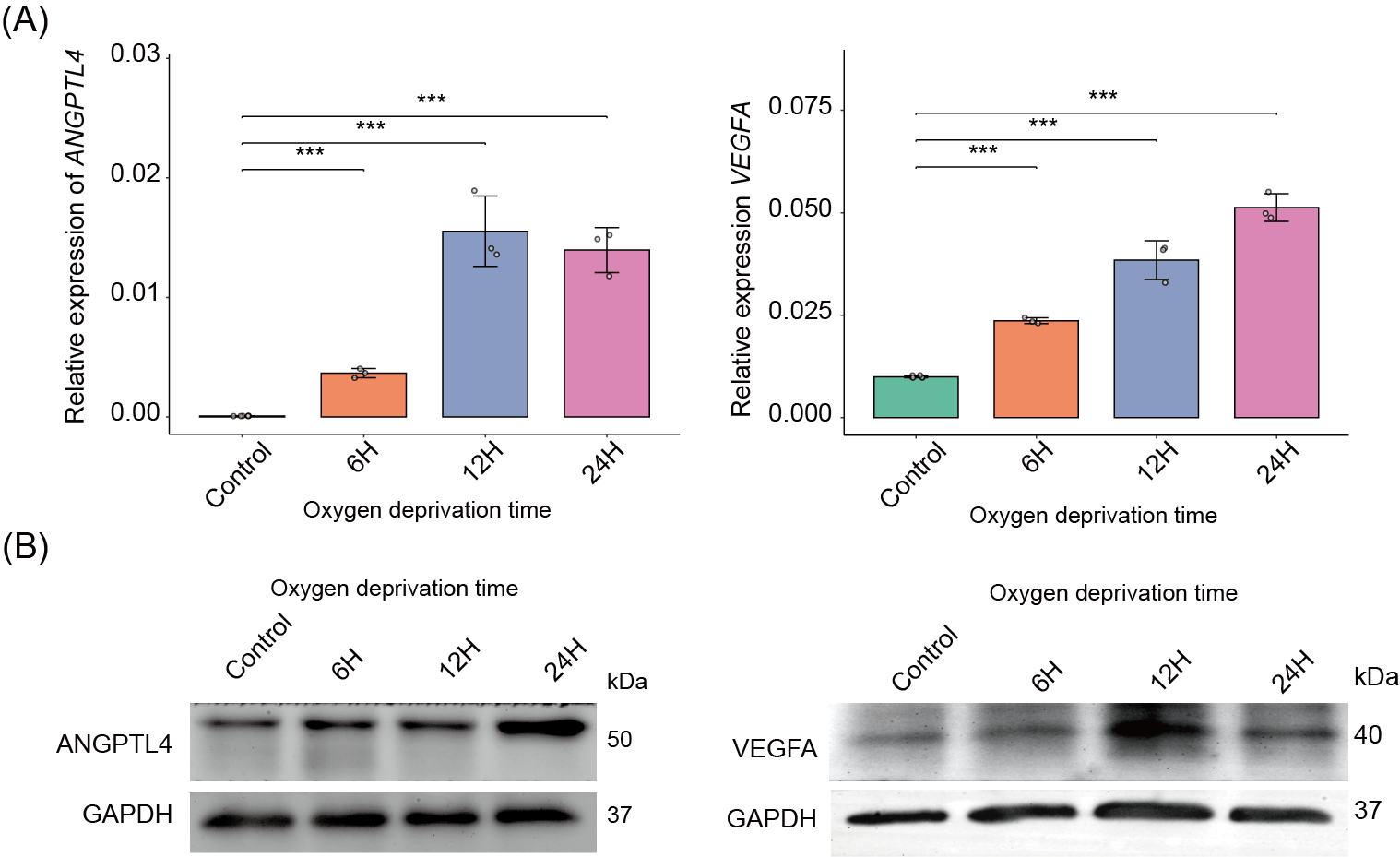


**Figure S4 Hypoxia induces time-dependent upregulation of ANGPTL4 and VEGFA in SMCs.** (A) Quantitative reverse transcription polymerase chain reaction (qPCR) analysis of angiopoietin-like 4 (*ANGPTL4*) and vascular endothelial growth factor A (*VEGFA*) messenger RNA (mRNA) expression in smooth muscle cells (SMCs) cultured under hypoxia for 0 h, 6 h, 12 h, and 24 h (n = 3 independent biological replicates per time point; one-way analysis of variance [ANOVA] followed by Dunnett's multiple-comparisons test versus 0 h). (B) Western blot analysis of ANGPTL4 and VEGFA protein expression in SMCs following hypoxic exposure for 0 h, 6 h, 12 h, and 24 h (n = 3 independent biological replicates per time point; ANOVA followed by Dunnett's multiple-comparisons test versus 0 h). Data are presented as the means ± SD; *** *p* < 0.001.
